# Automated segmentation of cardiomyocyte Z-disks from high-throughput scanning electron microscopy data

**DOI:** 10.1186/s12911-019-0962-1

**Published:** 2019-12-19

**Authors:** Afshin Khadangi, Eric Hanssen, Vijay Rajagopal

**Affiliations:** 1 Cell Structure and Mechanobiology Group, Department of Biomedical Engineering, Melbourne School of Engineering, The University of Melbourne, Melbourne, Australia; 2 Advanced Microscopy Facility, Bio21 Molecular Science and Biotechnology Institute, The University of Melbourne, Melbourne, Australia

**Keywords:** Cardiac ultrastructure, Image segmentation, Serial-block-face scanning electron microscopy, Focused ion-beam scanning electron microscopy, Computational biology

## Abstract

**Background:**

With the advent of new high-throughput electron microscopy techniques such as serial block-face scanning electron microscopy (SBF-SEM) and focused ion-beam scanning electron microscopy (FIB-SEM) biomedical scientists can study sub-cellular structural mechanisms of heart disease at high resolution and high volume. Among several key components that determine healthy contractile function in cardiomyocytes are Z-disks or Z-lines, which are located at the lateral borders of the sarcomere, the fundamental unit of striated muscle. Z-disks play the important role of anchoring contractile proteins within the cell that make the heartbeat. Changes to their organization can affect the force with which the cardiomyocyte contracts and may also affect signaling pathways that regulate cardiomyocyte health and function. Compared to other components in the cell, such as mitochondria, Z-disks appear as very thin linear structures in microscopy data with limited difference in contrast to the remaining components of the cell.

**Methods:**

In this paper, we propose to generate a 3D model of Z-disks within single adult cardiac cells from an automated segmentation of a large serial-block-face scanning electron microscopy (SBF-SEM) dataset. The proposed fully automated segmentation scheme is comprised of three main modules including “pre-processing”, “segmentation” and “refinement”. We represent a simple, yet effective model to perform segmentation and refinement steps. Contrast stretching, and Gaussian kernels are used to pre-process the dataset, and well-known “Sobel operators” are used in the segmentation module.

**Results:**

We have validated our model by comparing segmentation results with ground-truth annotated Z-disks in terms of pixel-wise accuracy. The results show that our model correctly detects Z-disks with 90.56% accuracy. We also compare and contrast the accuracy of the proposed algorithm in segmenting a FIB-SEM dataset against the accuracy of segmentations from a machine learning program called *Ilastik* and discuss the advantages and disadvantages that these two approaches have.

**Conclusions:**

Our validation results demonstrate the robustness and reliability of our algorithm and model both in terms of validation metrics and in terms of a comparison with a 3D visualisation of Z-disks obtained using immunofluorescence based confocal imaging.

## Introduction

The Z-disk (also known as Z-line or Z-band) forms the boundaries of sarcomeres, which are contractile components of striated muscles such as cardiac myocytes. Z-disks provide mechanical stability to cardiac and skeletal muscle [1]. α-actinin bridges (Z-bridges) crosslink the ends of thin filaments from adjacent sarcomeres, which are opposite in polarity. Z-disks stabilize the ends of these thin filaments, which are extended from Z-line towards the middle of sarcomere. These Z-lines are shown to be involved in very important processes of governing muscle homeostasis and stretch sensing. Moreover, several Z-disk proteins have recently been implicated in cardiomyopathies and cardiac diseases. Hence, an understanding of the structure of the Z-disk and changes to its topology may provide important insights into the development of cardiomyopathies [2].

Several microscopy studies have examined Z-disk topology and the distribution of other components (such as sarcoplasmic reticulum) relative to the Z-disk. Jayasinghe et al. [3] used confocal imaging to trace the organization of the Z-disks in rat ventricular myocytes. They reported that the Z-disks formed three-dimensional helical arrangements and exhibited dislocations through the myocyte volume. With the advent of serial block-face scanning electron microscopy (SBF-SEM), the limitation of targeted immunolabeling in confocal microscopy could be circumvented. The distribution of the Z-disks and its relation to other structures can be simultaneously visualized with these datasets. Studies such as [4, 5] have examined the distributions of other components relative to the Z-disks using SBF-SEM data. The authors investigated the three-dimensional structure of the intercalated disc and cardiac sarcoplasmic reticulum in relation to the Z-disks and reported that patterns of these structures are correlated with heart failure.

However, no studies to date have reported on image processing algorithms to extract Z-disks from SBF-SEM datasets. This is a significant challenge due to the inherently low contrast images that SBF-SEM produce. The z-disks occupy a relatively small fraction of the image volume when compared to other structures as well. A segmentation of the 3D organization of Z-disks from SBF-SEM data is also an important step towards building more detailed computational models of cardiomyocytes [6, 7]. Incorporating realistic distributions of Z-disks into these computational models would garner quantitative insights into the effect of changes to z-disk organization on the development of cardiomyopathies.

Here we propose to generate a 3D model of cardiac Z-disks based on image segmentation and edge detection. This work is an extension of our previous work [8], where we generated a publicly available automated workflow for segmenting single adult cardiac cells. We used a large volume serial block-face scanning electron microscopy (SBF-SEM) dataset to generate 3D meshes of myofibrils, mitochondria and nuclei. The dataset spans a volume of 215 μm × 46 μm × 60 μm at 50 nm isotropic voxel spacing and, as far as we know, it is the largest dataset of a single cardiac cell acquired by SBF-SEM. Here we aim to segment the Z-disks from this dataset to fuse the resulting segmentation with previously obtained results so that a more complete geometric model of a cardiomyocyte can be used to investigate the relationship between cardiomyocyte form and function [6].

SBF-SEM and other high throughput electron microscopy imaging techniques have the capability to provide a full three-dimensional view of large tissue blocks at resolutions ranging 10–50 nm, thus filling the gap between high resolution electron tomography and high contrast, confocal microscopy. However, manually segmenting the ultrastructural components from these large datasets is time-consuming, impractical and can lead to human-related errors [8]. On the other hand, machine learning methods such as convolutional neural networks [9], need pre-segmented and labelled datasets as annotations or ground-truth (GT) references, which also requires human intervention. We present an automated framework to segment cardiac Z-disks, with minimal human intervention where the intervention will only be necessary to validate the segmented results.

The rest of this paper is organized as follows: in section two, the methods and materials of the proposed image processing algorithm are outlined. Section three represents the output of the algorithm and the validation results. We also compare the robustness of the algorithm in segmenting Z-disks from a publicly available FIB-SEM dataset. Discussion is provided in section four where we compare the performance of the proposed method against the performance of a machine learning approach. In section five we conclude the paper and will discuss future work.

## Methods

In this section we first provide some details about the material and dataset used in our modelling. Next, we will outline the modules of the model in detail.

### Material

#### Tissue sample preparation

The tissue samples used for SBF-SEM imaging were prepared at the University of Auckland. All animal procedures followed guidelines approved by the University of Auckland Animal Ethics Committee (for animal procedures conducted in Auckland, Application Number R286).

The tissue blocks were dissected from the left ventricular free wall of 16 week-old male Sprague-Dawley rats. For more information regarding tissue preparation guidelines, the readers are encouraged to refer to “Tissue sample preparation” section of the previous work [8].

#### SBF-SEM

After the samples were collected, the orientation of muscle fibers was determined using microCT (GE nanotom microCT, TrACEES platform, The University of Melbourne) images of the whole resin block. The block was then trimmed to a square block face of 1 mm^2^ and 300 μm deep with fibers running perpendicular to the future cutting face. The sample was imaged in low vacuum mode (50 mbar) at 3 kV, 0.1 nA current using a backscattered detector. The acquisition pixel size was set at 10 nm for X and Y [8].

**Fig. 1:**
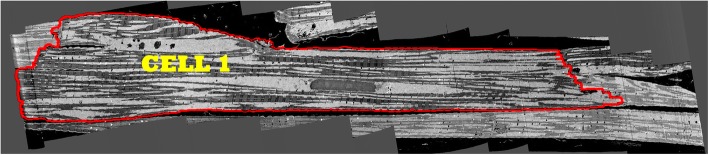
The outline of cell 1 (red contour) along with parts of the second cell that is adjacent to it in the bottom right

An example slice from the tissue block is shown in Fig. 1. For this tissue block, 1019 serial sections of 50 nm thickness were acquired. The data was binned to an isotropic voxel size 50 nm × 50 nm × 50 nm [8]. The stack contains 600 sections with 466× 2156 pixels in each section. As shown in Fig. 1, the stack contains two cells in which “cell 1” is outlined by a red contour. We aim to generate a model based on cell 1, since it contains the largest volume of heart cell structure information as the block captures the entire cell cross section and its length. Nevertheless, the proposed segmentation algorithm does segment the Z-disks in the entire image stack.

#### Image processing

**Fig. 2:**
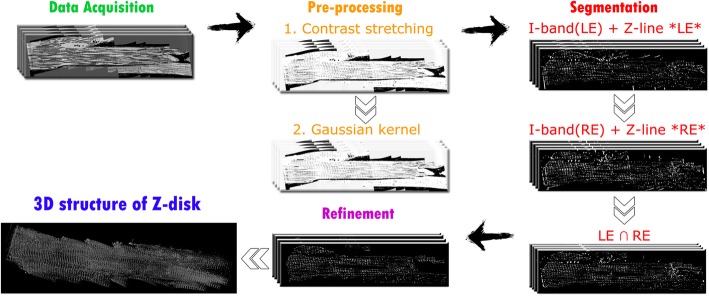
Schema of main image processing modules, corresponding sub-modules and 3D structure of Z-disk as output of model rendered in ImageJ

We propose an image processing framework to segment the Z-disks, by which we can generate a 3D model of them. Figure 2 represents the schema of the proposed image processing module. This framework comprises of three modules including pre-processing, segmentation and refinement. After the data is acquired, the pre-processing is performed. Then its outputs are treated in segmentation step. Finally, the segmentation results are refined in step three. These modules are performed on MATLAB R2017b, and for 3D reconstruction we have used ImageJ. The dataset (*.MRC format) was first loaded into ImageJ, and then image sequences were exported in TIFF format for further image processing to be performed on MATLAB.

#### Pre-processing

Pre-processing is known as one of the important tasks in pattern recognition and computer vision. Aside from intrinsic problems of datasets such as speckles, noise etc., they might contain information which are not necessary or required in our model. This extra information in the data could increase the complexity and computational cost. We utilize pre-processing to reduce noise in the data. Pre-processing also performs “feature selection” and “size reduction”, since it is manipulating the orientation of the feature space towards target space [10].

In our dataset, the image intensity distributions of the nuclei (*μ* = 71.17, *σ* = 12.53), myofibrils (*μ* = 55.57, *σ* = 6.19), I-bands (*μ* = 24.87, *σ* = 8.25), and Z-disks (*μ* = 63.57, *σ* = 11.07) are very similar, where “ ” and “ *σ* ” refer to the mean and standard deviation of the intensities, respectively.

**Fig. 3:**
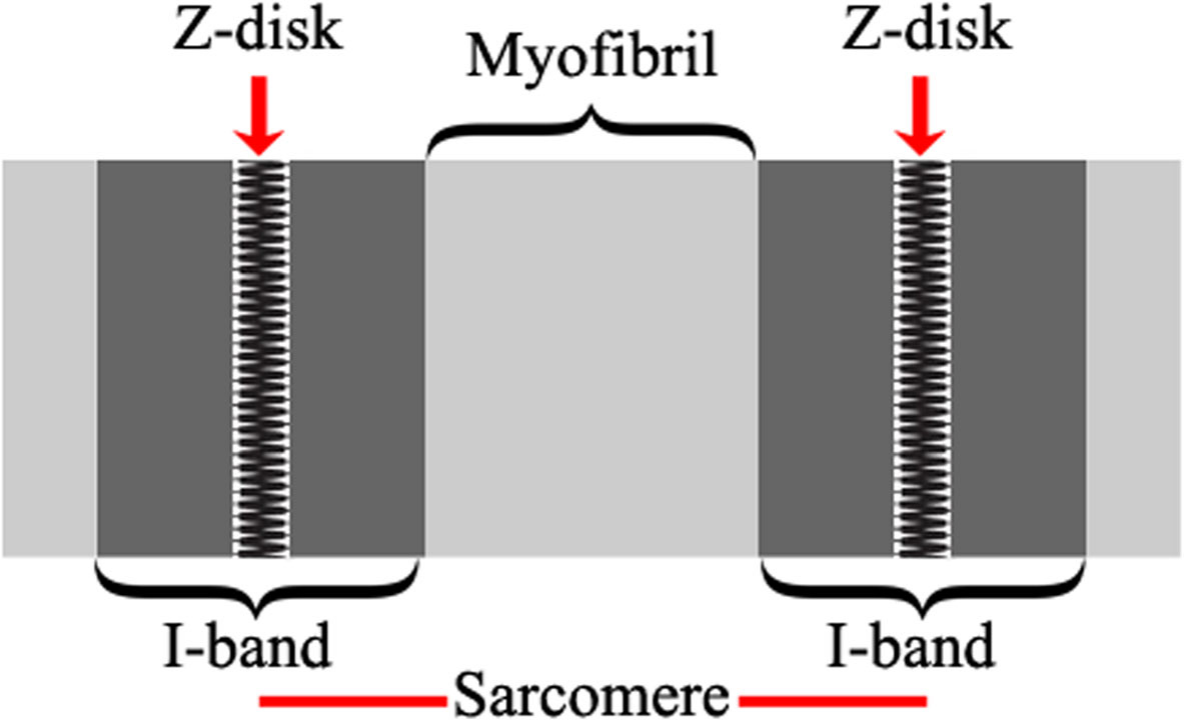
Structure of one sarcomere, alongside the definitions of I-band and Z-disk. The reader should note that this structure is only suited to represent our dataset, in which the M-band is not discoverable, i.e. myofibrils are located between two I-bands in our dataset

Figure 3 shows a schema of the structure of one sarcomere, the locations of corresponding I-bands and the Z-lines. These intensity distribution properties form the building blocks for the segmentation algorithm.

We perform local contrast stretching (LCS) which equalizes the contrast of the image to differentiate pixels in the I-band from the remainder of the sarcomere pixels. The LCS is conducted using a sliding kernel across the image and adjusting the central pixel by Eq. (1). The equation boosts the appearance of large-scale light-dark transitions [11]:

1 $$ {I}_{output}=255.\left[{I}_{input}-\mathit{\min}\right]/\left(\mathit{\max}-\mathit{\min}\right) $$

where,

*I*_*output*_ is the intensity level for the output pixel after LCS.

*I*_*input*_ is the intensity level for the input pixel (input data).

*max* and *min* are the maximum and minimum values for intensity level (gray level in our case) in input image, respectively.

**Fig. 4:**
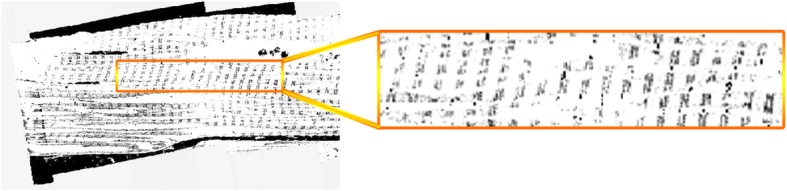
Output of local contrast stretching with max = 59 and min = 0

**Fig. 5:**
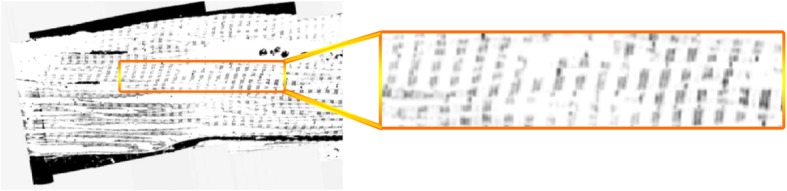
Output of Gaussian smoothing kernel with σ = 1.2

Since our aim is to localize and isolate the I-bands and Z-disks, we chose the values of *max* = 59 and *min* = 0 heuristically. The maximum value is very close to the average of the means of two intensity distributions corresponding to myofibrils and Z-disks: *μ*_*average* : *M* & *Z*_ = 59.57. This implies that the resulting image after LCS would help differentiate myofibrils and the Z-disks from I-bands, simultaneously. After LCS is performed, a Gaussian kernel with standard deviation of *σ* = 1.2 is used to smooth the output of LCS. Figures 4 and 5 represent the sample outputs of LCS and Gaussian smoothing, respectively.

#### Segmentation

As can be seen in Figs. 3 and 4, we observe a repeating pattern of vertical stripes which are specific to the nature of Z-disks. The Sobel operator is widely used for edge detection. The result of the Sobel operator is the image intensity gradient vector or the norm of this vector. The Sobel operator has two main advantages: (1) it has some smoothing effect on inherent random noise in the image;(2) it is a differential form of two rows or two columns, so the elements of the edge on both sides are enhanced, thus improving the edge quality and intensity [12, 13].

We perform a Sobel edge detection using 3 × 3 kernels as follows:

2 $$ {T}_x=\left|\begin{array}{ccc}-1& 0& +1\\ {}-2& 0& +2\\ {}-1& 0& +1\end{array}\right|\ {T}_y=\left|\begin{array}{ccc}-1& -2& -1\\ {}0& 0& 0\\ {}+1& +2& +1\end{array}\right| $$

where *T*_*x*_ and *T*_*y*_ are horizontal and vertical templates to be convolved with the image of interest.

Considering the patterns of I-bands and Z-disks as discussed above, we propose to employ the vertical Sobel operator templates to segment these components. The structure of the I-band consists of two vertical edges at the right and left ends of the I-band and the Z-disk is centered between these two edges. Hence, we can obtain a segmentation of Z-disks by first segmenting the I-band edges. However, we shall see that due to the value of *max* = 59 in Eq. (1), which is a trade-off between the mean values of intensity distributions of myofibrils and Z-disks, segmenting the edges of I-bands to be differentiated from myofibrils results in differentiation of Z-disks from I-bands.

We define Right Edge (RE) and Left Edge (LE) of I-band as its right and left borders, respectively (RE and LE are denoted in Fig. 2). The template operators of RE and LE are defined as follows:

3 $$ {T}_{RE}=\left|\begin{array}{ccc}-1& 0& +1\\ {}-2& 0& +2\\ {}-1& 0& +1\end{array}\right|\ {T}_{LE}=\left|\begin{array}{ccc}+1& 0& -1\\ {}+2& 0& -2\\ {}+1& 0& -1\end{array}\right| $$

**Fig. 6:**
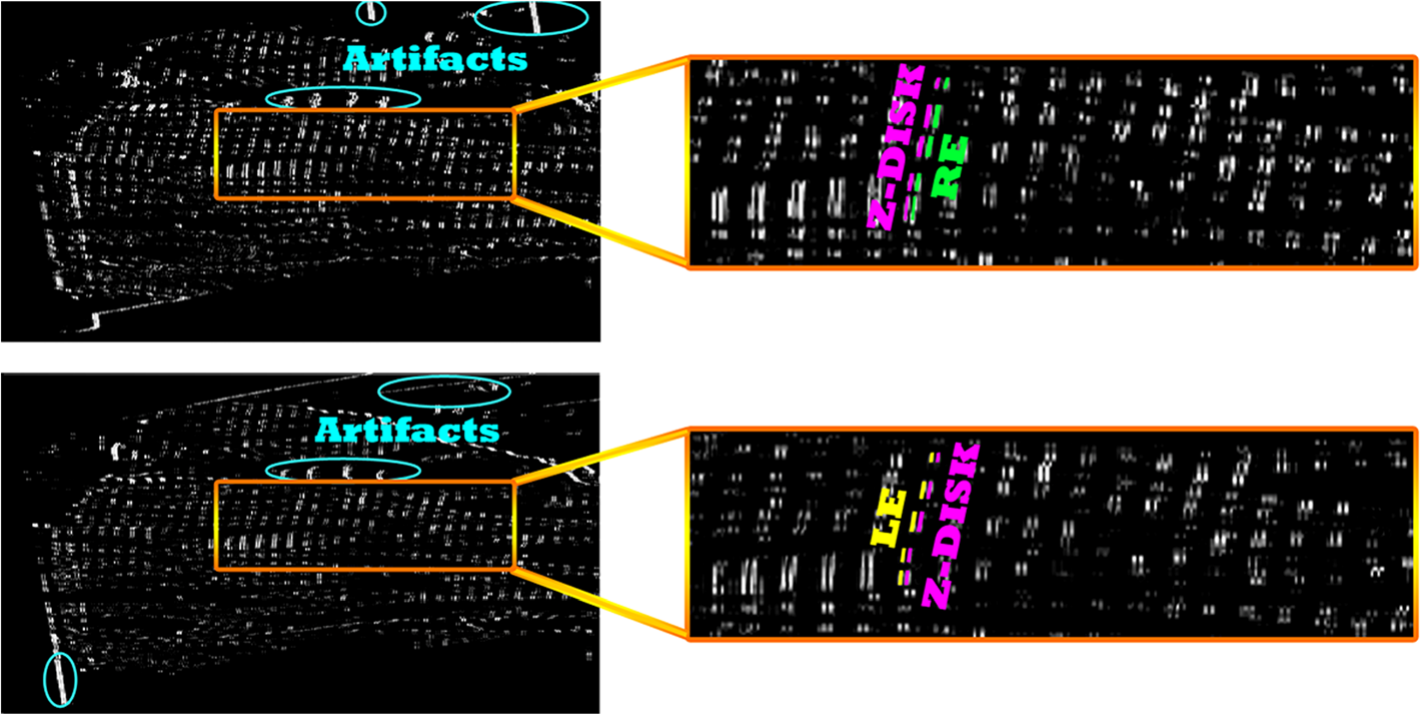
Top: output of convolution by T_RE, RE is highlighted in green color; Bottom: output of convolution by T_LE, LE is highlighted in yellow color; Z-disk and artifacts are shown in magenta and blue, respectively

To segment the right and left edges of I-bands, we convolved the corresponding templates with the output images from the pre-processing step. Figure 6 shows the output of these convolutions, where we have defined ***set*** of right and left edges as $\mathbf{\mathcal{R}}$ and $\mathbf{\mathcal{L}}$, Z-disks as $z$ and artifacts as **Θ** which are defined by Eq. (4) and (5):

4 $$ \mathcal{R}\cup z\cup \boldsymbol{\Theta} ={T}_{RE}\circledast I $$

5 $$ \mathcal{L}\cup z\cup \boldsymbol{\Theta} ={T}_{LE}\circledast I $$

where, ⊛ denotes two-dimensional signal processing convolution operation, and $I$ is the output image sequences from pre-processing step. From Fig. 6 we observe that the result of convolution is segmentation of Z-disks alongside right and left edges of I-bands, respectively. The RE and LE are located at the right side and left-side of Z-disks in Fig. 6, top and bottom, respectively. From the definitions provided in Eqs. (4) and (5), we seek to find set $z$ by first finding the ***intersection*** between the left sides of (4) and (5) by Eq. (6), and then removing the artifacts **Θ.** This step is performed in refinement module. Eq. (6) has the form as follows:

6 $$ \mathbf{\mathcal{z}}\cup \boldsymbol{\Theta} =\left\{\mathbf{\mathcal{R}}\cup z\cup \boldsymbol{\Theta} \right\}\cap \left\{\mathbf{\mathcal{L}}\cup \mathbf{\mathcal{z}}\cup \boldsymbol{\Theta} \right\} $$

**Fig. 7:**
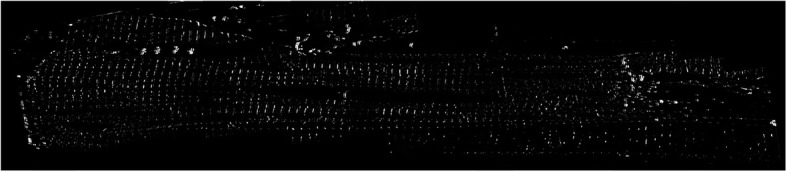
Sample output of intersection operation applied to images shown in Fig. 6

Equation (6) proves to be correct due to our definitions about $\mathbf{\mathcal{R}}$ and $\mathbf{\mathcal{L}}$ where $\mathbf{\mathcal{R}}\cap \mathbf{\mathcal{L}}=\varnothing$. The outputs of Eq. (6) applied to both images of Fig. 6, are shown in Fig. 7. The procedure presented above is applied to all the image slices after the preprocessing step, which involved in 1200 convolutions and 600 intersection operations.

#### Refinement

**Fig. 8:**
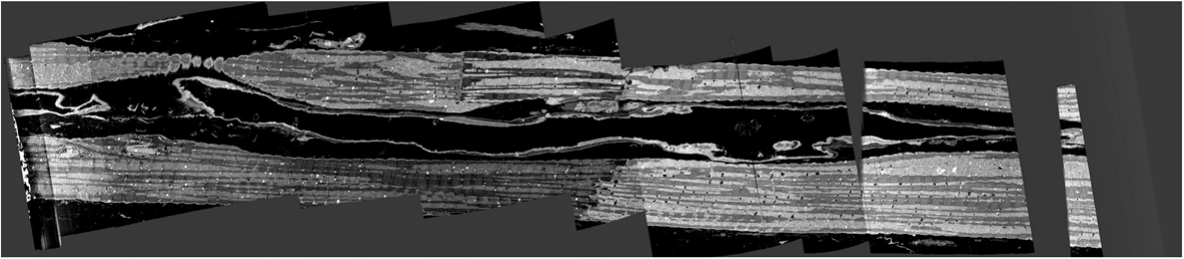
A sample of image sequence where more artifacts could arise due to cross-sectional drastic changes in intensities

Figure 7 shows a sample output of the intersection operation that was applied to images shown in Fig. 6. Some of the artifacts highlighted in Fig. 6 are also present in Fig. 7. To remove these artifacts, we isolate the regions where we are sure the Z-disks are present, i.e. between the Lower Membrane Edge (LME) and Upper Membrane Edge (UME). These edges are very hard to track through the image sequence because their shape and position can drastically change between any two images. Hence, the correct and reliable detection of these edges is vital to obtain reliable results. Figure 8 shows an example of an image slice that illustrates the challenge of finding the UME and LME.

To refine the segmented images, we propose to isolate Z-disk zones by finding LME and UME by convolving the images using horizontal templates of the Sobel operator as follows:

7 $$ {T}_{LME}=\left|\begin{array}{ccc}-1& -2& -1\\ {}0& 0& 0\\ {}+1& +2& +1\end{array}\right|\ {T}_{UME}=\left|\begin{array}{ccc}+1& +2& +1\\ {}0& 0& 0\\ {}-1& -2& -1\end{array}\right| $$

Considering the longitudinal orientation of the cells in the image sequences as shown in Fig. 1, we observe that after horizontal convolution operations are performed, the resulting segments would also contain some edges due to intensity changes between borders of mitochondria and myofibrils as well. However, due to lower intensity differences between mitochondria and myofibrils, as compared to that between mitochondria/myofibrils and the background (black spot), the resulting edges are very weak in terms of width and intensity.

Moreover, by convolving images with these templates, we obtain a set of fixed patterns of edges which pertain to the difference between background (black) and grey area in Fig. 1. In addition to the above, some of the artefacts have the form of densely packed clusters which do not pertain to Z-disks. These are shown in lower blue ellipses in Fig. 6.

To overcome the first issue above, we employed an erosion operator with a structuring element of 2 × 2 matrix of ones to erode the resulting images. This preserves the target edges of interest, since the edges corresponding to the first issue are too weak when compared to target edges of interest. The second issue related to fixed patterns of edges are simply removed by finding their coordinates. Finally, to remove the densely packed clusters, we employed a consecutive operation of erosion and dilation with structuring elements of 5 × 5 matrix of ones to first isolate these clusters and then dilating them to be removed from images.

**Fig. 9:**
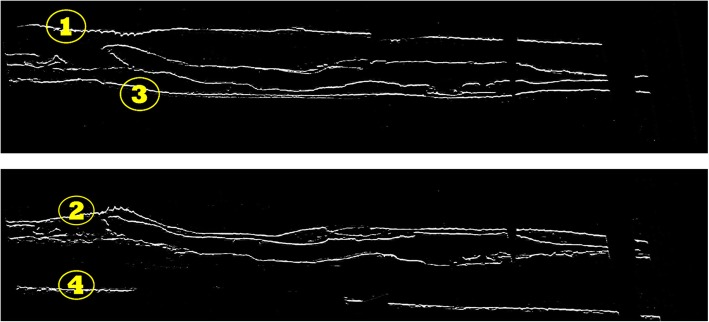
Resulting fine edges after the issues discussed in refinement section are resolved. Top: UME; Bottom: LME

After the initial step is performed to resolve the above issues arising from convolution, the resulting fine edges are presented in Fig. 9. As shown, more edges are obtained for this image because of significant changes in the structure of the cell. Moreover, some discontinuities are present in the edges. We are only interested in extracting edges highlighted by yellow circles.

According to Figs. 8 and 9, the target area where Z-disks are present, is the area between edge (1) of the top image and edge (2) of the bottom image in Fig. 9, as well as the area between edge (3) of the top image and edge (4) of the bottom image in Fig. 9. We perform another refinement procedure by defining a rule-based edge extraction method. To find the area between edge (1) and edge (2), and the area between edges (3) and (4) in Fig. 9, we propose the following rules to isolate Z-disks:

1. (*i*^*L*^, *j*^*L*^) = *find* (*I*_*LME*_(*i*, *j*) > 0) *i*, *j* ≤ *size*(*I*_*LME*_)
2. *define vector Ψ*_21 × 1_; |Φ| = (*i*^*L*^, *j*^*L*^)
3. Ψ_*LME*_(:) = *I*_*LME*_(*i*^*L*^ − 10 : *i*^*L*^ + 10, *j*^*L*^ + 1)
4. Λ = *find*(Ψ_*LME*_ > 0), 1)
5. *if* Λ is *empty* : *go to step* 6;
  $$ else:\Phi =\left[\Phi; \left[{i}^L+\Lambda -11,{j}^L+1\right]\right];{i}^L={i}^L+\Lambda -11 $$
6. *j*^*S*^ = *find*(*I*_*LME*_(*i*, *j*), 2) > 0; *i*^*L*^ − 20 ≤ *i* ≤ *i*^*L*^ + 20
  $$ if\kern0.50em {j}^S-{j}^L<10:\kern0.5em \Phi =\left[\Phi; \left[{i}^L,{j}^L+1\right]\right]; $$
  $$ else:\Phi =\left[\Phi; \left[\left\lfloor \frac{i-{i}^L}{\ {j}^S-{j}^L}\times \mathcal{J}+{i}^L\right\rfloor, \mathcal{J}\right]\right],{j}^L\le \mathcal{J}\le {j}^S;{j}^L={j}^S $$
7. *j*^*L*^ + = 1; *loop* 3 − 6 *until j*^*L*^ ≤ *size*(*I*_*LME*_, 2)
8. *I*_(2)_ = *I*_*LME*_; *I*_(0)_(:) = 0; *I*_(2)_(Φ) = 255

The definitions above illustrate necessary steps leading to isolating edge (2) in Fig. 9.

For the rest of the edges, the same rules apply, where steps 4 and 5 are modified to find the initial edge tracking point. The rule base for edge (2) of LME is interpreted as follows:

1. Find the pixel coordinates of *I*_*LME*_, where *I*_*LME*_(*i*^*L*^, *j*^*L*^) > 0, starting from the *i* = *j* = 1, and then shuffling through the columns and then incrementing through rows of the pixel coordinates. This will find the left tail of edge (2) in Fig. 9. This step could be interpreted as seeding the initial coordinates (*i*^*L*^, *j*^*L*^) of target edge of interest.
2. We define *Ψ*_21 × 1_ as a column vector to scan through a radius of 10 pixels around the neighborhood of current coordinate and store them temporarily. A matrix Φ is defined to store coordinates of edge of interest.
3. Scan through 10 pixels above and below the current row coordinate of the next column and store them in *Ψ*.
4. Find the first index of the elements of vector *Ψ*, where Ψ_*LME*_ > 0 and store in Λ. This is mainly because we are only interested in finding the upper edge (only for the case of edge (1) or (2). By contrast, for the case of edges (3) and (4) this is changed to finding the last element since we are interested in finding lower edge).
5. There are some regions where Λ could be empty i.e. edges are disjointed and there is no edge present in that coordinate, such as the case of edge (4) in LME in Fig. 9. In this case we propose to use one of the approaches presented in step 6. Step 6 isolates target regions since these discontinuities might arise from problems where some of the Z-disks could be excluded from the isolation regions. Otherwise, if the Λ is not empty, we will add the new coordinates to edge matrix Φ.
6. Find the first element of the left tail from next distant chunk as *j*^*S*^ by shuffling area of radius 20 centered at *i*^*L*^. If the longitudinal distance between *j*^*S*^ and previous *j*^*L*^ is less than 10 (the values of 20 for radius and 10 for longitudinal distance are chosen heuristically), then the edge coordinates will continue same as previous coordinates located at right tail of previous chunk, resulting in horizontal edge. Threshold value of 10 here ensures Z-disks will be preserved in regions where discontinuities might be extensive as the case of edge (4) in Fig. 9. If the longitudinal distance is equal to or greater than 10, we will use a linear transformation between *j*^*L*^ and *j*^*S*^ i.e. a line-shaped edge connecting these two coordinates together. This will help preserve Z-disk information which exceeds the performance of case where we only stuck into continuing horizontal line. This is proved to be effective for the case of edge (4) in Fig. 9, where in an extreme condition, the cell area is adjacent to grey area in Fig. 8, where horizontal edge will result in great loss, because in this scenario, some of the Z-disks will fall below the edge, thus will be excluded.
7. Shuffle through the columns by incrementing *j*^*L*^. Loop through steps 3 to 6 until the stopping criterion is met which is defined as *j*^*L*^ ≤ *size*(*I*_*LME*_, 2).
8. After the coordinates of edges are found we will pad the resulting edge to a zero-binary image *I*_(0)_ as size as original image *I*_*LME*_. The resulting edge is defined as *I*_(2)_.

**Fig. 10:**
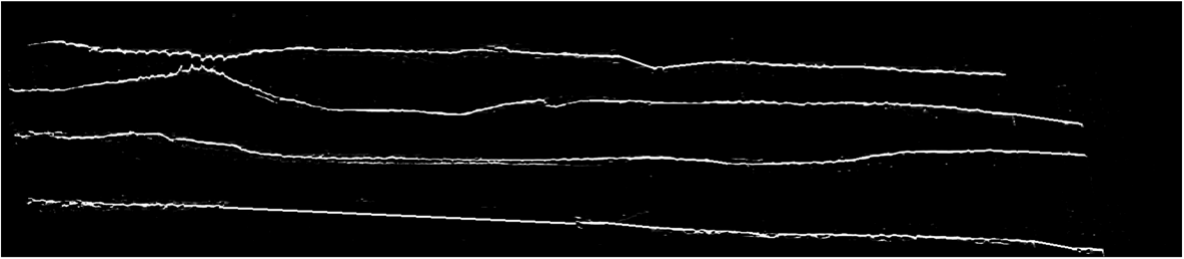
Resulting contiguous fine edges after performing refinement stage

**Fig. 11:**
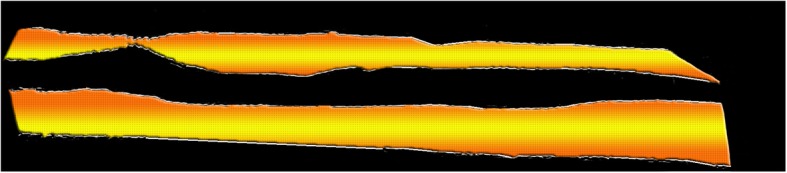
Shaded area corresponding to Z-disks region. The coordinates of these regions are utilized to isolate Z-disks by extracting these coordinates from $z\cup \boldsymbol{\Theta}$ outlined in Eq. (6)

**Fig. 12:**
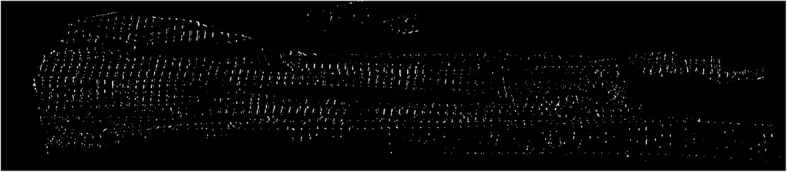
Fine segmentation result of Z-disks after performing refinement on sample image shown in Fig. 7

This framework is applied to extract and pad the edges *I*_(1)_, *I*_(3)_ and *I*_(4)_ along with edge *I*_(2)_ to *I*_(0)_. The result of applying this framework on images of Fig. 9, is shown in Fig. 10. After we obtained the contiguous fine edges illustrated in Fig. 10, we keep the information within the shaded area as shown in Fig. 11 which correspond to Z-disks and remove the remaining information. The output of refinement step applied to the sample image of Fig. 7 is shown in Fig. 12. After the refinement module was performed we obtained image sequences corresponding to segmented Z-disks. To ensure that we capture all the necessary information required for a fine 3D model, the segmentation results were refined further by implementing steps outlined in the image processing module on the other two orthogonal planes of the collected volume data, as we did in previous work for segmenting myofibrils, mitochondria and nuclei [8]. In the next section we generate a 3D model of Z-disks and validate the segmentation results.

## Results

### Visualization

**Fig. 13:**
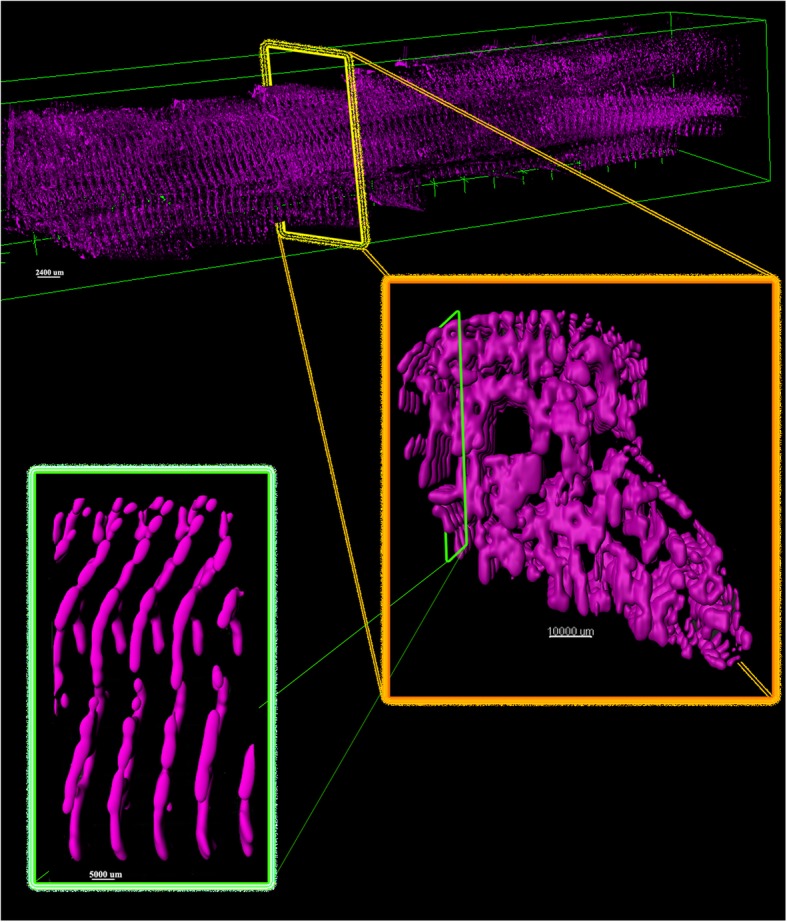
Generated 3D model of cardiac Z-disks. The volumes are rendered in ImageJ and Imaris

For visualization purposes, we have used 3D plugin of ImageJ [14] and Imaris v9.2.1 software available at http://bitplane.com. After the obtained segmented image sequences were imported to the software, we rendered the volume by using its 3D plugin. Figure 13 represents the output of the segmentation model as generated 3D model of cardiac Z-disks from different angles of view, rendered in ImageJ.

### Validation

After the set of ground-truth (GT) images are determined, we have compared them with the final segmented Z-disks. Table 1 shows the validation results in terms of accuracy, sensitivity and specificity. To define these metrics, we have categorized the individual pixels in both sets of GT and model outputs into four classes as TP, TN, FP and FN defined as follows:

- TP (True-Positive): it is a set of pixels segmented as Z-disks by model which match manual annotations.
- TN (True-Negative): it is the set of pixels that are not segmented as Z-disks in model, which match the background of manual annotations.
- FP (False-Positive): it is a set of pixels segmented as Z-disks by model, which are not present as Z-disks in manual annotations.
- FN (False-Negative): it is a set of pixels which are not segmented as Z-disks by model, while are segmented as Z-disks in manual annotations.
- Considering definitions above, validation metrics are defined as follows:

**Table 1:** Model validation metrics and their corresponding values

Model validation	Metrics		
	Accuracy	Specificity	Sensitivity
Values %	90.56%	89.27%	92.23%

8 $$ accuracy=\frac{TP+ TN}{TP+ TN+ FN+ FP} $$

9 $$ specificity=\frac{TN}{TN+ FP} $$

10 $$ sensitivity=\frac{TP}{TP+ FN} $$

The corresponding values of validation metrics are outlined in Table 1. The values of metrics show that the algorithm is robust, and we can rely on the generated 3D model of the cardiac Z-disks.

**Fig. 14:**
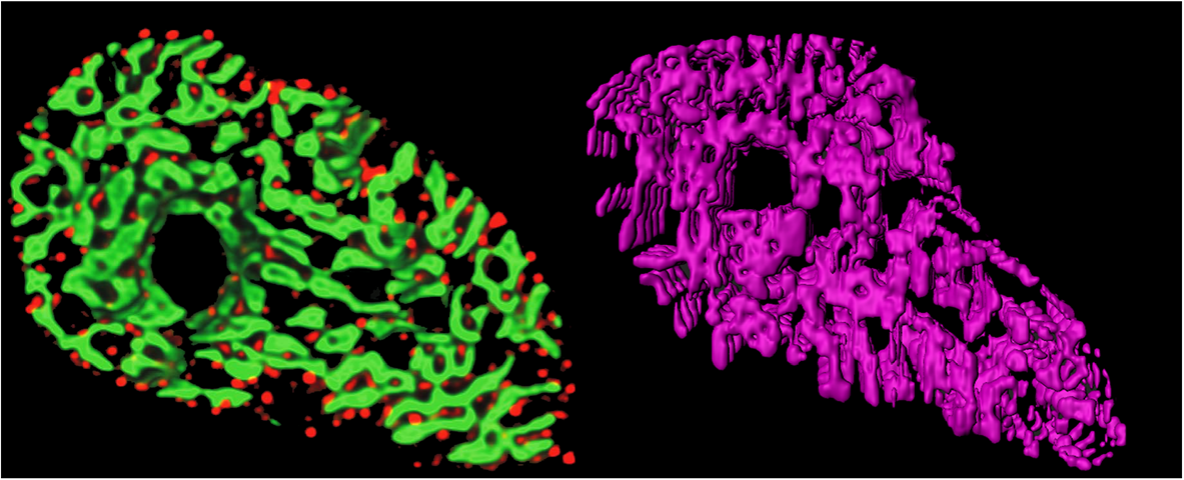
Comparison between obtained results and ground-truth 3D structure of cardiac Z-disks; left: 3D rendering of Z-disks (marked in green) from stained myocyte with antibody to α-actinin [15]; right: rendered 900–1000 segmented sequences of proposed model (surface accuracy 400 um)

In addition to validating results with manual annotations, we have made a comparison between our results and a previously published 3D structure of Z-disks rendered from rat ventricular myocyte [15]. Soeller et al. had performed double labeling with an antibody to α-actinin to visualize Z-line structure. The authors had obtained interesting results which showed Z-disks in ventricular myocytes are not flat but curved with frequent bifurcations that form connections to adjacent Z-disks. Figure 14 illustrates a comparison between longitudinal distribution of Z-disks obtained by Soeller et al. (Fig. 14, left, Z-disks are marked in green), and slices 900 to 1000 of segmented Z-disks by our model rendered in 3D (Fig. 14, right). This illustration adds extra degree of reliability to our model along with the promising validation metric values provided in Table 1.

### Performance in segmenting FIB-SEM data

**Fig. 15:**
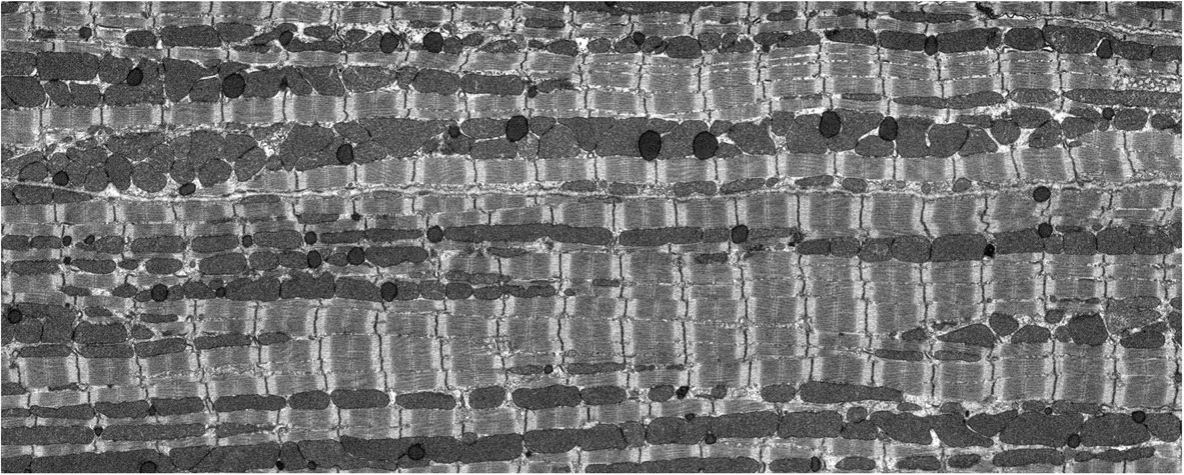
A sample slice from FIB-SEM dataset used for evaluation of proposed segmentation method

**Fig. 16:**
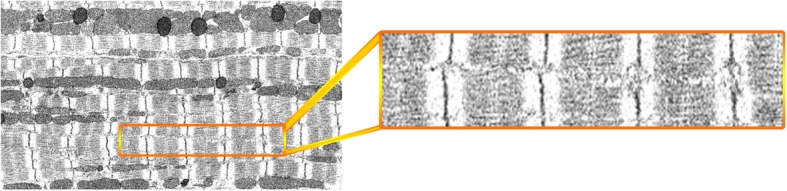
Output of local contrast stretching with max = 160 and min = 0

**Fig. 17:**
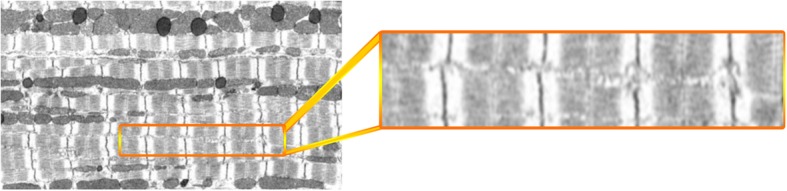
Output of Gaussian smoothing kernel with σ = 2

To test whether our proposed method [16] can be applied to datasets other than that acquired using University of Melbourne Teneo Volumescope machine, we used the method to segment Z-disks on another published FIB-SEM dataset of cardiomyocyte ultrastructure [17]. We applied the same procedure as applied to SBF-SEM data to process the FIB-SEM dataset except for the pre-processing stage. For the LCS, we chose the values of *max* = 160 and *min* = 0 in Eq. (1). The maximum value is very close to the average of the means of the two intensity distributions corresponding to myofibrils and Z-disks: *μ*_*average* : *M* & *Z*_ = 160.32. After LCS was performed, we utilized a Gaussian kernel with standard deviation of *σ* = 2 to smooth the output of LCS. Figures 15, 16 and 17 represent a sample slice of the FIB-SEM data, the outputs of LCS and Gaussian smoothing, respectively.

To validate the segmentation results obtained from processing the new dataset, we manually annotated three randomly chosen slices from a total 792 slices, each having 2922 × 1166 pixels. We used the same validation metrics as used for the SBF-SEM dataset. The corresponding values of validation results are outlined in Tables 2 and 3, where the first represents the results of applying the same refinement step used for SBF-SEM data and the second shows the results after a disk-shaped structuring element with diameter of 1 was used for dilating the obtained Z-disks masks. As shown, adding dilation to the refinement step improved the validation results. This is mainly because of the spatial resolution at which the Z-disk structures were imaged in the two different datasets.

**Table 2:** Validation metrics on the performance of the proposed algorithm on a fib-sem dataset of cardiac ultrastructure

Model validation	Metrics		
	Accuracy	Specificity	Sensitivity
Values %	78.42%	99.74%	57.10%

**Table 3:** Validation metrics for proposed algorithm with a z-disk dilatation step on a fib-sem dataset of cardiac ultrastructure

Model validation	Metrics		
	Accuracy	Specificity	Sensitivity
Values %	90.56%	99.74%	80.85%

Segmentation of Z-disks in the FIB-SEM data using MATLAB required less than 20 min to segment the whole volume (792 slices in Z direction). Table 3 shows that the proposed model can segment about 90% of the pixels in the new dataset correctly. However, the sensitivity or true positive rate was lower by approximately 10% in comparison with the validation results of SBF-SEM data. Our analysis showed that the proposed model segments membranes of mitochondria as Z-disks in some regions and misses the Z-disks where the corresponding structures deviate from the characteristic vertical line.

## Discussion

Machine learning approaches adapt their underlying parameters to the data and learn to extract valuable information from data structures as features [9, 18]. After such features are extracted, a classifier can be used to classify the data into desirable target classes.

Machine learning is widely used with applications in biological image analysis and many software packages are available with applications in biological image analysis that utilize such automated methods for applications including pixel classification. Among commercial software packages used for processing SBF-SEM data, the most commonly used tools are Digital Micrograph (DM; Gatan, UK), Amira (FEI, UK) and Imaris (Bitplane, Switzerland). Some of the widely used open source packages also utilised for processing such 3D data volumes include IMOD [19], ImageJ [14] and ilastik [20]. Such software packages provide a variety of image analysis tools ranging from annotating, image enhancement, transform, denoising, annotating tools and 3D rendering for visualisation purposes to embedded trainable segmentation tools which use machine learning methods to segment data.

Ilastik provides a user-friendly pixel classifier module where the user can import the raw images, select predefined features or add more custom features, define labels and annotate the images for training. The software offers a batch processing module which can be used to generate predictive segmentation masks for a given dataset. Moreover, the module provides a feature selection tool in which the software performs a grid search to suggest features to the user that would enable reliable segmentation of specific labels in the dataset. The module includes 37 pre-defined features in total and a user can select any arbitrary subset of them to train ilastik on the dataset.

We experimented with several pixel classification tasks within ilastik 1.3.0 to segment Z-disks in the SBF-SEM and FIB-SEM datasets. Our experiments showed that ilastik was not able to segment the Z-disks accurately within the SBF-SEM dataset. This is likely due to the low contrast between Z-disks and the remaining components of the cell in the dataset and also because of the few pixel-widths with which the Z-disk segments were represented in the data (1–2 pixels in width). However, in the FIB-SEM data the width of Z-disk segments ranges from 5 to 7 pixels and we therefore found that ilastik performed better on this dataset.

All our experiments were implemented on HP Z440 Workstation with Intel® Xeon® CPU E5–1620 @ 3.5 GHz, with 32 GB Memory. We trained the ilastik classifier to segment FIB-SEM data with 4 distinct experiments, where we used 1, 2, 3 and 8 ground-truth images for training the classifier which took approximately 0.5, 0.72, 1.10 and 4.25 h to train the model. These images were chosen randomly and none of the 3 manually segmented (validation set) images were used during the training. One user spent 1.5 h on average to manually annotate the background and Z-disk labels on each image which took about 12 h in total to finish the labeling. Moreover, to segment the whole volume we used the batch processing module of ilastik and it took further 10 h to process the remaining 784 images.

**Fig. 18:**
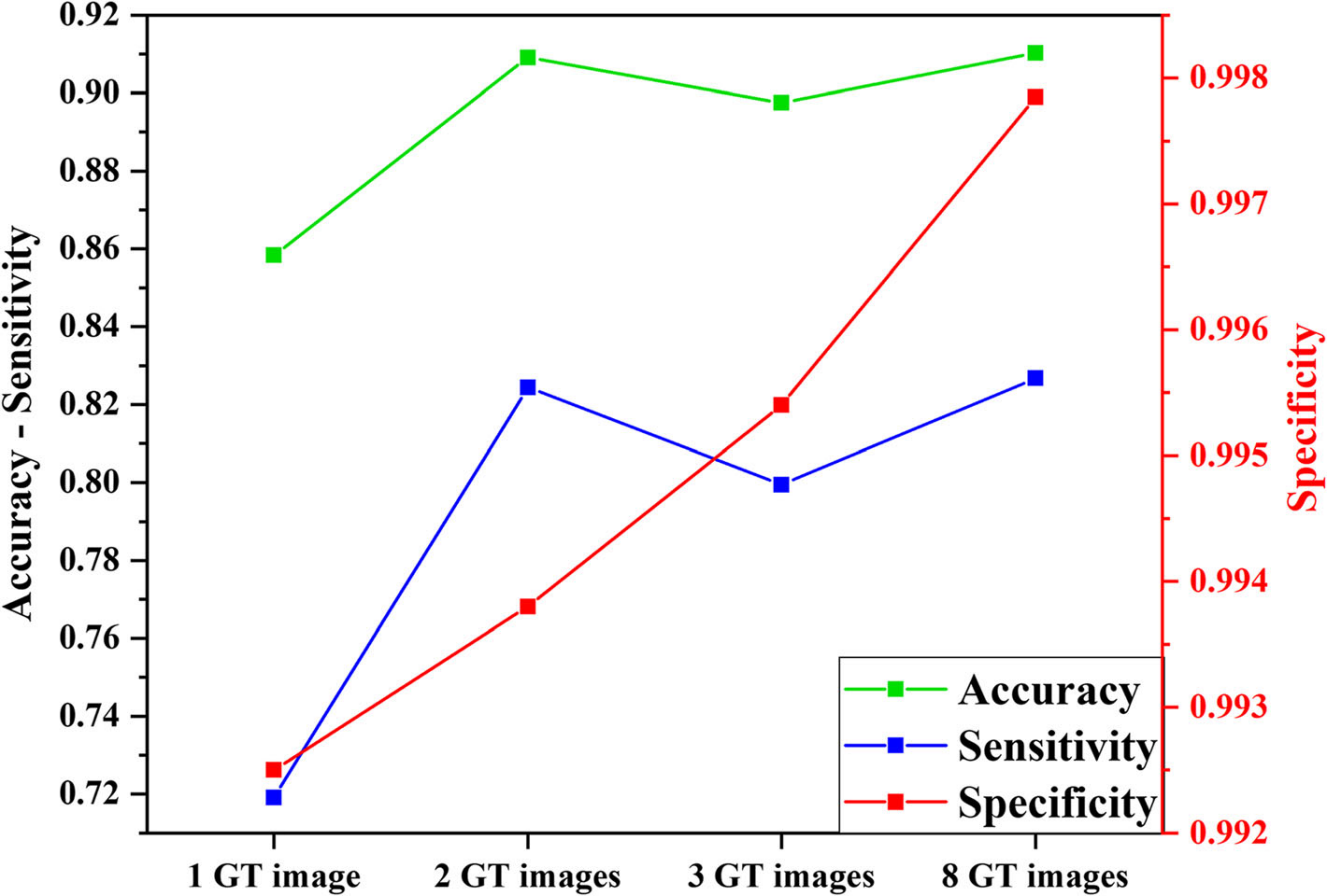
Comparison between validation results of segmenting Z-disk structures on FIB-SEM data using ilastik. GT = ground-truth

Figure 18 shows the results obtained by our experiments on ilastik to segment Z-disk structures on FIB-SEM data. As shown, by increasing the number of ground-truth images for training, the validation metrics improve as the experiment with 8 ground-truth images achieves maximum validation accuracy, sensitivity and specificity. Noteworthy is the trend in specificity values as we increase the training dataset from 1 ground-truth image to 8 ground-truth images. The change in specificity shown in red in Fig. 18 can be attributed to the relatively small number of pixels representing Z-disks when compared to non-Z-disk, background pixels. The classifier learns to favor the true negative rate or specificity much more, as there is a total of approximately 10 million non-Z-disk pixels within three image slices, while there are only approximately 100,000 Z-disk pixels (~ 1% of one slice of FIB-SEM data). As shown in Fig. 18, increasing the number of ground-truth images results in minor improvement. Table 4 shows the comparison between the results of our proposed method and ilastik on segmenting Z-disks from the FIB-SEM data. Ilastik outperforms our proposed model in segmenting Z-disks by a small percentage.

**Table 4:** Comparison between the validation results of proposed model and ilastik on segmenting Z-disks in FIB-SEM data

Method	Metrics		
	Accuracy	Specificity	Sensitivity
Proposed method	90.56%	99.74%	80.85%
ilastik	91.03%	99.78%	82.67%

We analyzed the results on the validation dataset in more depth by overlaying the output masks in ImageJ. As shown in Table 4, ilastik can segment the Z-disk structures correctly by about 82% whereas this value is about 80% for our proposed model. Our analysis showed that ilastik performs better than our proposed model in segmenting the Z-disks in terms of the accuracy of the Z-disk cluster width. The width of segmented structures using ilastik was about 6 pixels on average, whereas this value was 5 pixels on average using the proposed method after the dilation step. Increasing the width of the dilatation structural element to compensate for the reduced accuracy of the segmented Z-disk width using the proposed algorithm did not improve results. This is because the pixels representing false positive Z-disks using the proposed algorithm also increased when the structural element width was increased.

In summary, ilastik failed to segment the Z-disks on SBF-SEM data where the Z-disk structures were 1-pixel wide or at most 2 pixels wide, whereas our proposed method was able to segment this dataset. Ilastik outperformed our proposed model on segmenting the FIB-SEM dataset, however, it took more than 22 h to segment the whole volume, whereas it took only 20 min (~40x faster) for our proposed method to segment these structures in the FIB-SEM volume with comparable results as shown in Table 4.

## Conclusion

In this paper we have developed an automated framework to segment cardiac Z-disks from SBF-SEM stack data and consequently generate a 3D model of this component of cardiac myocytes from the left ventricle of a rat heart. Our validation results demonstrate the robustness and reliability of our algorithm and model both in terms of validation metrics and in terms of a comparison with a 3D visualisation of Z-disks obtained using immunofluorescence based confocal imaging.

We proposed to segment the Z-disks using image processing methods that minimise manual annotation. Approaches like machine-learning require annotations and labeling prior to training. This is a very time-consuming task for the case of Z-disks. In addition, manual segmentations are not reliable always due to human error and are known to hinder effective diagnosis in the context of medical image processing [21], especially where the details are very subtle as in the case of the Z-line. Secondly, we performed experiments to test the robustness of our algorithm by segmenting Z-disks from an SBF-SEM and a FIB-SEM dataset. We also compared its performance relative to the open source software ilastik. The experiments showed that ilastik outperformed the proposed method in segmenting Z-disks on the FIB-SEM dataset but was not able to segment 1–2 pixel-width Z-disks in the SBF-SEM dataset. Ilastik also took ~40x more time than our proposed method to prepare training data (manual annotation) and segment the volume.

We seek to merge the resulting structure of Z-disks into the results of the previous work [8], where we generated 3D finite model of ultrastructures including mitochondria, myofibrils and nuclei. A more complete model that included Z-disks, myofibrils, mitochondria and nuclei could be used to study the relationship between cardiac cell form and function in greater detail.

## Data Availability

We offer our research code and data for research use at www.github.com/CellSMB/.

## Abbreviations

BOMP: Biological Optical Microscopy Platform
FIB-SEM: Focused ion-beam scanning electron microscopy
FN: False-negative
FP: False-positive
GT: Ground-truth
LCS: Local contrast stretching
LE: Left edge
LME: Lower membrane edge
RE: Right edge
SBF-SEM: Serial block-face scanning electron microscopy
TN: True-negative
TP: True-positive
UME: Upper membrane edge
